# Artificial intelligence-guided quantitative coronary CT assessment to rule-in or rule-out myocardial ischaemia

**DOI:** 10.1093/ehjci/jeag094

**Published:** 2026-04-13

**Authors:** Putri Annisa Kamila, Nick S Nurmohamed, Ibrahim Danad, Ruurt A Jukema, Pieter G Raijmakers, Roel S Driessen, Michiel J Bom, Pepijn van Diemen, Gianluca Pontone, Daniele Andreini, Hyuk-Jae Chang, Richard J Katz, Andrew D Choi, Paul Knaapen, Jeroen J Bax, Alexander van Rosendael, the CREDENCE and PACIFIC-1 Investigators, Ran Heo, Hyung-Bok Park, Hugo Marques, Wijnand J Stuijfzand, Jung Hyun Choi, Joon-Hyung Doh, Ae-Young Her, Bon-Kwon Koo, Chang-Wook Nam, Sang-Hoon Shin, Jason Cole, Alessia Gimelli, Muhammad Akram Khan, Bin Lu, Yang Gao, Faisal Nabi, Mouaz H Al-Mallah, Ryo Nakazato, Randall C Thompson, James J Jang, Michael Ridner, Chris Rowan, Erick Avelar, Philippe Généreux, Guus A de Waard

**Affiliations:** Department of Cardiology, Leiden University Medical Center, Albinusdreef 2, Leiden 2333 ZA, The Netherlands; Faculty of Medicine, Universitas Brawijaya, Malang, Indonesia; Department of Cardiology, Amsterdam UMC, Vrije Universiteit Amsterdam, Amsterdam, The Netherlands; Department of Vascular Medicine, Amsterdam UMC, University of Amsterdam, Amsterdam, The Netherlands; Department of Cardiology, Radboud University Medical Center, Nijmegen, The Netherlands; Department of Cardiology, Amsterdam UMC, Vrije Universiteit Amsterdam, Amsterdam, The Netherlands; Department of Radiology and Nuclear Medicine, Amsterdam UMC, Vrije Universiteit Amsterdam, Amsterdam, The Netherlands; Department of Cardiology, Amsterdam UMC, Vrije Universiteit Amsterdam, Amsterdam, The Netherlands; Department of Cardiology, Amsterdam UMC, Vrije Universiteit Amsterdam, Amsterdam, The Netherlands; Department of Cardiology, Amsterdam UMC, Vrije Universiteit Amsterdam, Amsterdam, The Netherlands; Department of Perioperative Cardiology and Cardiovascular Imaging, Centro Cardiologico Monzino IRCCS, Milan, Italy; Department of Biomedical, Surgical and Dental Sciences, University of Milan, Milan, Italy; Department of University Cardiology and Cardiac Imaging, IRCCS Ospedale Galeazzi Sant’Ambrogio, Milan, Italy; Division of Cardiology, Severance Cardiovascular Hospital and Severance Biomedical Science Institute, Yonsei University College of Medicine, Yonsei University Health System, Seoul, South Korea; Division of Cardiology, The George Washington University School of Medicine, Washington, DC, USA; Division of Cardiology, The George Washington University School of Medicine, Washington, DC, USA; Department of Cardiology, Amsterdam UMC, Vrije Universiteit Amsterdam, Amsterdam, The Netherlands; Department of Cardiology, Leiden University Medical Center, Albinusdreef 2, Leiden 2333 ZA, The Netherlands; Heart Center, Turku University Hospital and University of Turku, Turku, Finland; Department of Cardiology, Division of Heart and Lungs, Utrecht University, Utrecht University Medical Center, Utrecht, The Netherlands

**Keywords:** coronary computed tomography angiography, atherosclerosis, coronary artery disease, artificial intelligence, coronary ischaemia

## Abstract

**Aims:**

To evaluate the ability of artificial intelligence-based quantitative CT (AI-QCT) parameters, diameter stenosis, percent atheroma volume (PAV) and average lumen area (ALA) to rule-in or rule-out ischaemia.

**Methods and results:**

This *post-hoc*, vessel-level analysis included patients with suspected coronary artery disease from the computed tomographic evaluation of atherosclerotic determinants of myocardial ischaemia (CREDENCE) (612 patients; 1727 vessels) and PACIFIC-1 (208 patients; 612 vessels) studies who underwent CCTA and invasive fractional flow reserve (FFR). In addition to diameter stenosis, PAV and ALA were evaluated as key predictors of ischaemia. We report abnormal FFR prevalence based on these variables and define rule-out (<15% ischaemia prevalence, defer further testing), rule-in (>75% prevalence, ischaemia highly likely; further testing typically unnecessary), and intermediate risk (15–75%, consider additional functional assessment). PAV and ALA were dichotomized using median values derived from the CREDENCE cohort (14.7% and 3.9 mm^2^) and validated in PACIFIC-1. In CREDENCE, all vessels with 1–24% stenosis were ruled-out. Among vessels with 25–49% stenosis, 74% met rule-out criteria, while 26%, characterized by large PAV and small ALA, were intermediate risk. Within the proposed framework vessels with 50–69% stenosis were classified as intermediate risk. For 70–99% stenosis, 93% met rule-in criteria, except a small subset with small PAV and large ALA. In PACIFIC-1, 86% of vessels with <50% stenosis were ruled-out, and 61% of those with 50–99% stenosis were ruled-in.

**Conclusion:**

A simplified framework incorporating AI-QCT parameters including diameter stenosis, PAV (>14.7%), and ALA (<3.9 mm^2^), stratifies myocardial ischaemia risk. Most non-obstructive lesions can be ruled-out, while most stenoses >70% are reliably ruled-in. This practical approach enhances the diagnostic utility of CCTA and streamlines clinical decision-making.

## Graphical Abstract

**Graphical Abstract:**
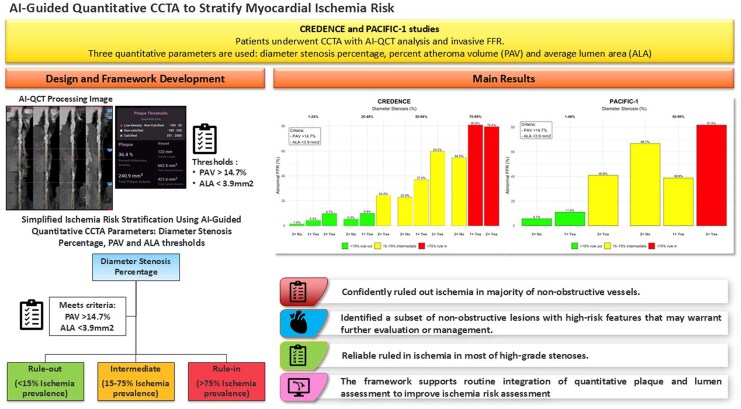

## Introduction

Coronary artery disease (CAD) remains a leading cause of global morbidity and mortality. In symptomatic patients, assessment of myocardial ischaemia helps to clarify the cause of chest pain and guide further diagnostic evaluation and management.^1,2^ Current guidelines recommend coronary computed tomography angiography (CCTA) as an effective first-line test for evaluating patients with suspected CAD.^3,4^ Traditionally, the ischaemic risk of coronary arteries assessed by CCTA has been defined by the presence or absence of obstructive coronary artery stenosis (defined as ≥50% luminal narrowing).^5^ However, many obstructive stenoses do not result in ischaemia, while some non-obstructive lesions are functionally significant.^6–8^ This discordance underscores the limitations of a stenosis-based approach and highlights the need for a more comprehensive evaluation of CAD.

Advancements in CCTA, particularly with artificial intelligence (AI)-guided quantitative analysis, enable more granular evaluation of coronary morphology and disease burden. Incremental to percent diameter stenosis, which captures only the focal point of luminal narrowing, AI-derived quantitative plaque and lumen metrics offer a more comprehensive assessment of the anatomical and physiological contributors to ischaemia.^9,10^ Among these, percent atheroma volume (PAV), a marker of total plaque burden normalized to vessel size, has been associated with the presence of ischaemia. Likewise, average lumen area (ALA), derived from lumen volume adjusted for vessel length, has demonstrated strong predictive value for identifying hemodynamically significant lesions.^11,12^

However, for these quantitative features to be used in clinical practice, specific cut-offs must be defined. Applying these thresholds to CCTA should generate a post-test likelihood of ischaemia that is low enough to rule out the need for further testing, or high enough to rule it in. Previous analyses from the CREDENCE and PACIFIC-1 studies demonstrated that plaque burden and lumen size are associated with myocardial ischaemia^12,13^; however, specific thresholds for clinical use were not established. In this study, we define and validate thresholds for PAV and ALA, and apply them together with diameter stenosis, in a simplified three-metric framework. This approach allows vessels to be categorized as low, intermediate, or high risk for ischaemia, with the goal of supporting more informed and efficient diagnostic evaluation and clinical management.

## Methods

### Study population

This study is a *post-hoc* analysis of the CREDENCE (computed tomographic evaluation of atherosclerotic determinants of myocardial ischaemia) study (*n* = 612 patients; 1727 coronary arteries)^13^ and the PACIFIC-1 [comparison of coronary computed tomography angiography, single photon emission computed tomography, positron emission tomography, and hybrid imaging for diagnosis of ischaemic heart disease determined by fractional flow reserve (FFR)] study (*n* = 208 patients; 612 coronary arteries).^14^ The CREDENCE study (conducted across 23 centres between 2014 and 2017), enrolled patients without known CAD who had symptoms suspicious of CAD and were referred for non-emergent invasive coronary angiography (ICA). The PACIFIC-1 study, conducted from 2012 to 2014, included symptomatic patients without known CAD who had an intermediate pre-test likelihood of CAD, scheduled for clinically indicated, non-emergent ICA. In both studies, all participants underwent CCTA and ICA with unbiased three-vessel FFR measurements, as the reference standard of ischaemia.^15,16^

### CCTA imaging protocols

In CREDENCE, coronary CTA was performed using single- or dual-source CT scanners from multiple vendors, all with at least 64 detector rows. In PACIFIC-1, coronary CTA was performed using a 256–detector row CT scanner (Philips Brilliance iCT, Philips Healthcare). All images were acquired in accordance with the guidelines provided by the Society of Cardiovascular Computed Tomography.^17^

### AI-based quantitative computed tomography analysis

The AI-based analysis of CCTA scans from both studies was conducted using a validated and FDA-approved software platform (Cleerly Labs, Cleerly Inc., Denver, CO). This software employs a series of validated convolutional neural networks, including 3-dimensional U-Net and Visual Geometry Group variants, to perform automated image quality assessment, coronary segmentation and labelling, lumen and vessel contour evaluation, as well as quantitative plaque characterization.^18^ The reliability of AI-based quantitative computed tomography (AI-QCT) has been established through prior validation in multi-centre trials against expert consensus, quantitative coronary angiography, FFR, and intravascular ultrasound.^18–20^

The quantitative output of the AI-QCT analysis included stenosis parameters such as percent diameter stenosis, as well as vascular morphological features, including vessel volume, lumen volume, and vessel length. The ALA was derived by dividing the lumen volume by vessel length. Additionally, the algorithm provided detailed assessments of atherosclerotic plaque characteristics, including plaque volumes and plaque burden. Plaque volumes (measured in mm^3^) were quantified for each coronary lesion and subsequently aggregated to determine the total plaque volume at the segment, vessel, and patient levels. To account for variations in coronary artery size, plaque burden was normalized to vessel volume and expressed as PAV, calculated as (plaque volume/vessel volume) × 100%.

### ICA and FFR

In CREDENCE, ICA was performed in accordance with clinical indications and imaging standards. FFR measurements were obtained distal to stenoses in major coronary arteries and side branches (≥2.0 mm diameter) with luminal narrowing between 40% and 90%, during intracoronary or intravenous adenosine infusion. For lesions with ˂40% stenosis, FFR was performed if deemed necessary. The images were then analysed by an independent, blinded core laboratory for performance of quantitative coronary angiography and FFR reliability. In the PACIFIC-1, ICA was performed according to a standardized protocol, with at least 2 orthogonal views per evaluated segment.^14^ FFR was routinely measured in all major coronary arteries, regardless of visible stenosis, except in occluded or subtotal lesions (≥90%). Maximal hyperaemia was achieved using intracoronary (150 µg) or intravenous (140 µg/kg/min) adenosine. Angiographic images and FFR data were reviewed by experienced cardiologists, blinded to the non-invasive imaging results. In both studies, FFR was calculated as the ratio of mean distal intracoronary pressure to mean aortic pressure, with ischaemia defined by an FFR value ≤0.8.

### Ischaemia assessment by diameter stenosis, PAV and ALA

Quantitative coronary artery diameter stenosis was categorized as 0%, 1–24%, 25–49%, 50–69%, 70–99%, and 100% in accordance with the coronary artery disease-reporting and data system (CAD-RADS) 2.0.^21^ To ensure the generalizability of the findings, a two-stage approach was used: the primary quantitative (PAV and ALA) thresholds were first derived from the larger CREDENCE cohort to maximize statistical stability and subsequently tested on the independent PACIFIC-1 cohort for external validation. Lesions with 0% or 100% stenosis were excluded, as plaque characterization would not alter risk assessment in these cases.

PAV and ALA were dichotomized based on median values in the CREDENCE cohort (14.7% and 3.9 mm^2^, respectively), resulting in four subgroups: (i) small PAV, large ALA; (ii) small PAV, small ALA; (iii) large PAV, large ALA; and (iv) large PAV, small ALA. These pre-defined thresholds were then applied to the PACIFIC-1 population for external validation. Diameter stenosis in PACIFIC-1 was categorized as non-obstructive (1–49% stenosis) and obstructive (≥50% stenosis) due to lower available number of vessels. In addition, to improve comparability with the CREDENCE cohort, additional exploratory vessel-level analyses were performed using the same stenosis categories applied in CREDENCE (1–24%, 25–49%, 50–69%, and 70–99%).

**Figure 1:**
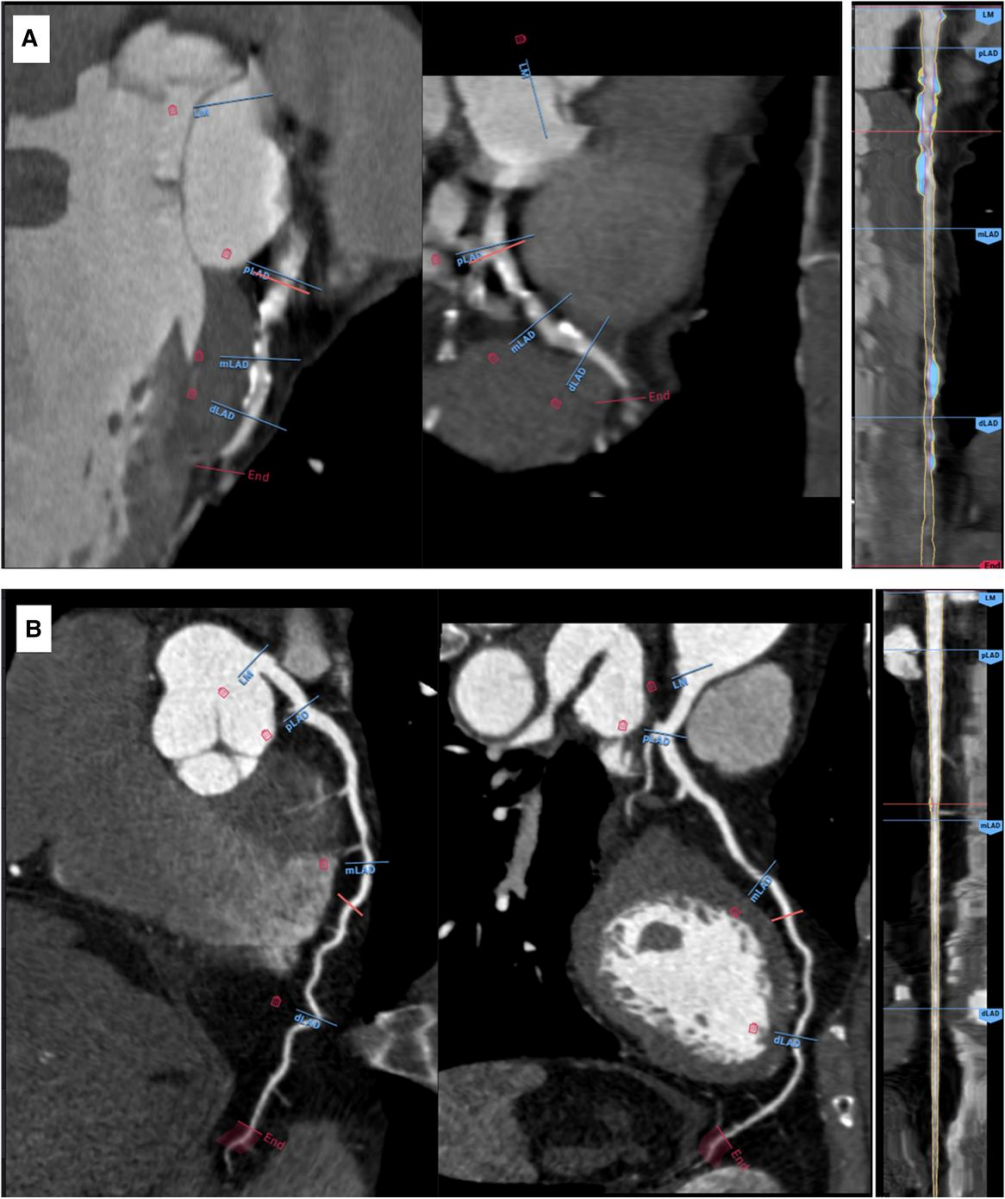
Example cases of coronary vessels analysed by AI-based quantitative computed tomography (AI-QCT). (*A*) Coronary CT angiography (CCTA) of the left anterior descending artery (LAD) from a 74-year-old man, analysed using AI-QCT, showing high PAV (34.7%) and a small ALA (2.52 mm^2^). (*B*) CCTA of the LAD from a 76-year-old man, analysed using AI-QCT, showing low PAV (9.57%) and a large ALA (4.53 mm^2^). Images used are provided courtesy of Cleerly and used with permission.

Further sensitivity analysis, alternative cut-offs for PAV and ALA were derived in the CREDENCE cohort using Youden’s index and subsequently applied to both the CREDENCE and PACIFIC-1 cohorts. Representative AI-QCT analysis is shown in *Figure 1A* and *B*, depicting coronary vessels with contrasting PAV and ALA.

### Ischaemia risk stratification: determining rule-out and rule-in groups

The thresholds for risk stratification were based on established clinical guidelines, prior studies and principles of diagnostic utility. A rule-out group for ischaemia was defined as a post-test prevalence of abnormal FFR <15%, indicating a low likelihood of ischaemia. This threshold reflects an established low-likelihood boundary used in diagnostic probability frameworks and is conceptually aligned with low-risk definitions applied in guideline-based risk assessment, although applied here after CCTA rather than as a clinical pre-test estimate. A rule-in group for ischaemia was defined as a post-test prevalence of abnormal FFR ≥75%, indicating a high likelihood of ischaemia at the vessel level. Coronary arteries with a post-test ischaemia prevalence between 15% and 75% were classified as intermediate risk, indicating residual diagnostic uncertainty and the potential need for additional functional assessment.^1,22,23^

### Statistical analysis

Baseline characteristics were summarized using descriptive statistics. Continuous variables were tested for normality using the Shapiro–Wilk test. Normally distributed variables were presented as mean ± standard deviation (SD), while non-normally distributed variables were reported as median with inter-quartile range (IQR). Comparisons of continuous variables between FFR ≤0.80 and FFR >0.80 were performed using the non-parametric Mann–Whitney *U* test. Categorical variables were expressed as counts and percentages and compared using χ^2^ or Fisher’s exact tests, as appropriate.

Analyses evaluating ischaemia risk were performed at the vessel level, consistent with FFR-based assessment for each coronary artery. Thresholds for PAV and ALA were primarily defined using the median values from the CREDENCE cohort and validated in PACIFIC-1. To determine the prevalence of ischaemia, vessels were first stratified by stenosis severity. Within each stenosis category, they were further sub-stratified into four groups based on the dichotomized PAV and ALA thresholds. The prevalence of abnormal FFR (≤0.80) was then calculated for each of these final subgroups.

Areas under the receiver-operating-characteristic curve (AUCs) were used to evaluate the incremental value of PAV and ALA over diameter stenosis for the prediction of abnormal FFR. Logistic regression was used to combine these parameters with diameter stenosis. As a sensitivity analysis, alternative cut-off values for PAV and ALA were derived in the CREDENCE cohort using Youden’s index and applied to both the CREDENCE and PACIFIC-1 cohorts. Diagnostic performance using Youden-derived thresholds was assessed using ROC curve analysis and compared with the primary median-based thresholds using the DeLong test. A *P*-value of <0.05 was considered statistically significant. All statistical analyses were performed using IBM SPSS Statistics Version 29.0.0.0 and R studio version 4.5.0.

## Results

### Patients

In the CREDENCE cohort, the mean age was 64.4 ± 10 years, and 69.9% of patients were male. The majority (71%) were symptomatic, and cardiovascular risk factors were common: 64% had hypertension, 30.4% had type 2 diabetes mellitus, and 50.3% had dyslipidaemia. In the PACIFIC-1 cohort, all patients were symptomatic, with a mean age of 58.1 ± 8.7 years; hypertension, type 2 diabetes mellitus, and dyslipidaemia were present in 46%, 16%, and 40% of patients, respectively. Additional baseline characteristics are detailed in *Table 1*.

**Table 1:** Patient characteristics in CREDENCE and PACIFIC-1

	CREDENCE (*n* = 612)	PACIFIC-1 (*n* = 208)
Age (years), mean (SD)	64.4 (10.0)	58.1 (8.7)
Male (%)	428 (69.9%)	132 (63.5%)
Hypertension, *n* (%)	394 (64.4%)	96 (46.2%)
Diabetes mellitus, *n* (%)	186 (30.4%)	33 (15.9%)
Dyslipidaemia, *n* (%)	308 (50.3%)	83 (39.9%)
Current smoking, *n* (%)	120 (19.6%)	99 (47.6%)
Familial history of CAD, *n* (%)	120 (19.6%)	107 (51.4%)
Symptoms		
Typical angina, *n* (%)	233 (38.1%)	71 (34%)
Atypical angina, *n* (%)	122 (19.9%)	80 (38.5%)
Non-cardiac chest pain, *n* (%)	74 (12.1%)	57 (27.4%)
Asymptomatic, *n* (%)	183 (29.9%)	0 (0)
Medication		
Aspirin, *n* (%)	360 (58.8%)	182 (87.5%)
Beta-blocker, *n* (%)	161 (26.3%)	135 (64.9%)
Statin, *n* (%)	357 (58.3%)	162 (77.9%)
Calcium antagonist, *n* (%)	189 (30.9%)	61 (29.3%)
Long acting nitrates, *n* (%)	49 (8%)	22 (10.6%)

*n*, number of patients; CAD, coronary artery disease.

### Incremental diagnostic value of PAV and ALA for detecting abnormal FFR

In CREDENCE, the AUC of diameter stenosis alone to diagnose abnormal FFR was 0.83 (95% CI: 0.81–0.86, *P* < 0.001). The addition of PAV significantly increased the AUC to 0.86 (95% CI: 0.83–0.88, *P* < 0.001). Further addition of ALA yielded another significant improvement to a final AUC of 0.87 (95% CI: 0.85–0.89; *P* < 0.001).

In the PACIFIC-1 cohort, a diameter stenosis alone yielded an AUC of 0.86 (95% CI: 0.82–0.89) for detecting hemodynamically significant ischaemia. Adding PAV improved it to 0.88 (95% CI: 0.85–0.92, *P* < 0.001). Incorporating ALA resulted in a final AUC of 0.88 (95% CI: 0.85–0.92, *P* > 0.5), with no significant difference compared with the two-metric model. The final three-metric model, nonetheless, remained significantly superior to the baseline model of stenosis alone (0.88 [95% CI: 0.85–0.92] vs. 0.86 [95% CI: 0.82–0.89], *P* < 0.001).

**Figure 2:**
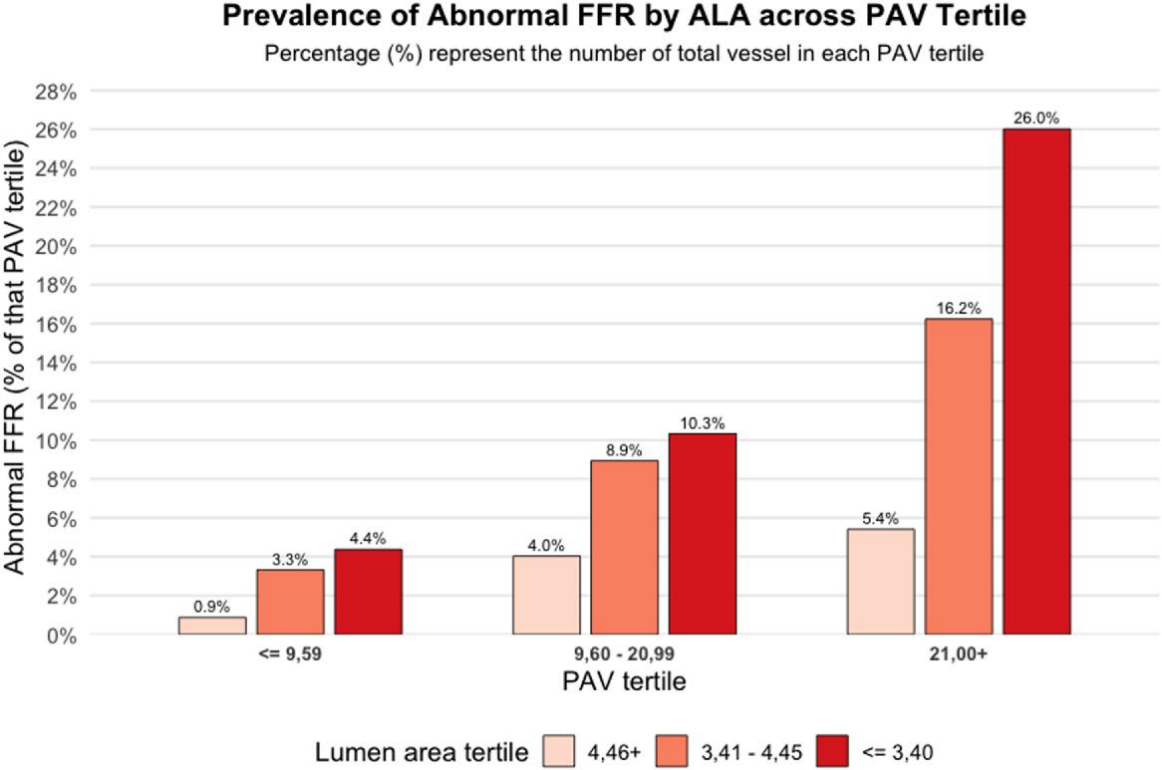
Correlation between PAV tertiles and ALA in relation to abnormal fractional flow reserve (FFR) prevalence in the CREDENCE study population. This Figure shows the prevalence of abnormal FFR across increasing PAV tertiles, stratified by ALA. As PAV increases (≤9.59%, 9.60–20.99%, >21.00%), the prevalence of abnormal FFR rises progressively. Within each PAV tertile, smaller ALA (≤3.40 mm^2^) are consistently associated with a higher prevalence of abnormal FFR compared with larger ALA (3.41–4.45 mm^2^ and >4.46 mm^2^), highlighting the additive diagnostic impact of ALA reduction.

The independent diagnostic value of PAV and ALA is presented in *Figure 2*. Increasing PAV relates with a higher prevalence of abnormal FFR, while smaller and larger lumen area significantly modifies this relationship.

### Diameter stenosis, PAV, and ALA for ruling-in/-out abnormal FFR

**Figure 3:**
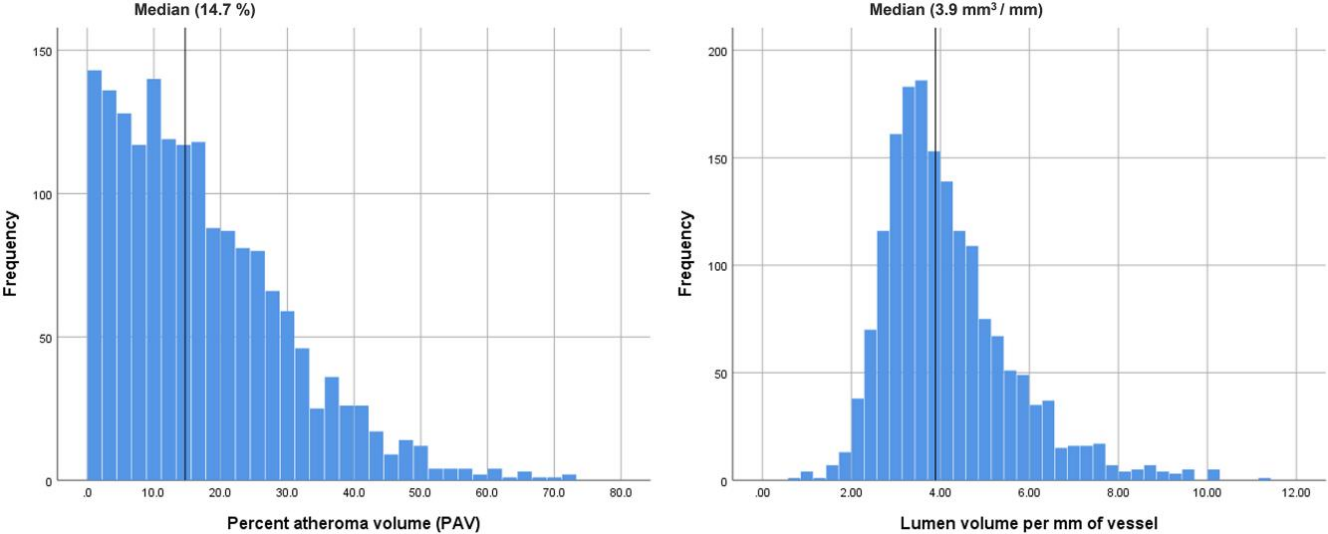
Distribution of PAV and ALA in the CREDENCE study population. This Figure illustrates the skewed distribution of PAV (left) and ALA (right). Median values (14.7% for PAV and 3.9 mm^2^ for ALA) were used as cut-off values in subsequent analyses.

In the CREDENCE (*n*: 1727 vessels), the median for PAV and ALA were 14.7% and 3.9 mm^2^, respectively. These values were used as thresholds and are herein referred to as ‘large’ (above median) and ‘small’ (below median) for simplicity. The distribution of PAV and ALA was left-skewed, as illustrated in *Figure 3*. After exclusion of vessels with 0% (*n* = 105) or 100% (*n* = 121) stenosis, and missing data (*n* = 11), 1490 vessels remained, with 348 (23.4%) having abnormal FFR. Ischaemia prevalence increased stepwise with stenosis severity (3.2% in 1–24% stenosis vs. 78.1% in 70–99% stenosis; *P* < 0.001, *Table 2*). Vessels with abnormal FFR had significantly higher median PAV (25.1% vs. 12.9%; *P* < 0.001) and smaller ALA (3.45 mm^2^ vs. 4.16 mm^2^; *P* < 0.001) compared with non-ischaemic vessels. When stratified by median thresholds, ischaemia prevalence was substantially higher in vessels with large PAV (36.4% vs. 9.1%; *P* < 0.001) and in those with small ALA (33.8% vs. 14.2%; *P* < 0.001).

**Table 2:** Per-vessel characteristics based on diameter stenosis, PAV, ALA, and FFR in CREDENCE

CREDENCE	Overall (*n* = 1490)	FFR >0.80 (*n* = 1142)	FFR ≤0.80 (*n* = 348)	*P*-value
Maximum lumen diameter stenosis				<0.001
1–24%	412	399 (96.8%)	13 (3.2%)	
25–49%	625	548 (87.7%)	77 (12.3%)	
50–69%	293	160 (54.6%)	133 (45.5%)	
70–99%	160	35 (21.9%)	125 (78.1%)	
PAV	15.4 (IQR 8.2; 24.9)	12.9 (IQR 6.89; 21.2)	25.1 (IQR 17.1; 33.3)	<0.001
<14.7%	712	647 (90.9%)	65 (9.1%)	
>14.7%	778	495 (63.6%)	283 (36.4%)	
ALA	3.94 (IQR 3.30; 4.92)	4.16 (IQR 3.40; 5.20)	3.45 (IQR 3.00; 4.12)	<0.001
>3.9 mm^2^	791	679 (85.8%)	112 (14.2%)	
<3.9 mm^2^	699	463 (66.2%)	236 (33.8%)	

*n*, number of vessels; FFR, fractional flow reserve; PAV, percent atheroma volume; ALA, average lumen area; IQR, inter-quartile range

These findings were validated in the PACIFIC-1 cohort (*n* = 485), where 28.5% of vessels were ischaemic (*Table 3*). Using the pre-specified PAV threshold of 14.7%, large PAV was associated with a more than six-fold higher prevalence of ischaemia (53.8% vs. 8.8%; *P* < 0.001). Similarly, an ALA below 3.9 mm^2^ was linked to a nearly three-fold increase in ischaemia prevalence (41.6% vs. 15.0%; *P* < 0.001).

**Table 3:** Per-vessel characteristics based on diameter stenosis, PAV, ALA, and FFR

PACIFIC-1	Overall (*n* = 485)	FFR >0.80 (*n* = 347)	FFR ≤0.80 (*n* = 138)	*P*-value
Maximum lumen diameter stenosis				<0.001
1–24%	247	233 (94.3%)	14 (5.7%)	
25–49%	96	66 (68.8%)	30 (31.3%)	
50–69%	74	39 (52.7%)	35 (47.3%)	
70–99%	68	9 (13.2%)	35 (47.3%)	
PAV %	12.3 (IQR 3.40; 25.1)	7.30 (IQR 0.2; 15.8)	28.3 (IQR 19.2; 41.3)	<0.001
<14.7%	273	249 (91.2%)	24 (8.8%)	
>14.7%	212	98 (46.2%)	114 (53.8%)	
ALA	3.84 (IQR 3.10; 5.54)	4.27 (IQR 3.26; 6.04)	3.29 (IQR 2.78; 3.99)	<0.001
>3.9 mm^2^	240	204 (85.0%)	36 (15.0%)	
<3.9 mm^2^	245	143 (58.4%)	102 (41.6%)	

*n*, number of vessels; FFR, fractional flow reserve; PAV, percent atheroma volume; ALA, average lumen area; IQR, inter-quartile range

### Risk stratification by diameter stenosis, PAV and ALA

**Figure 4:**
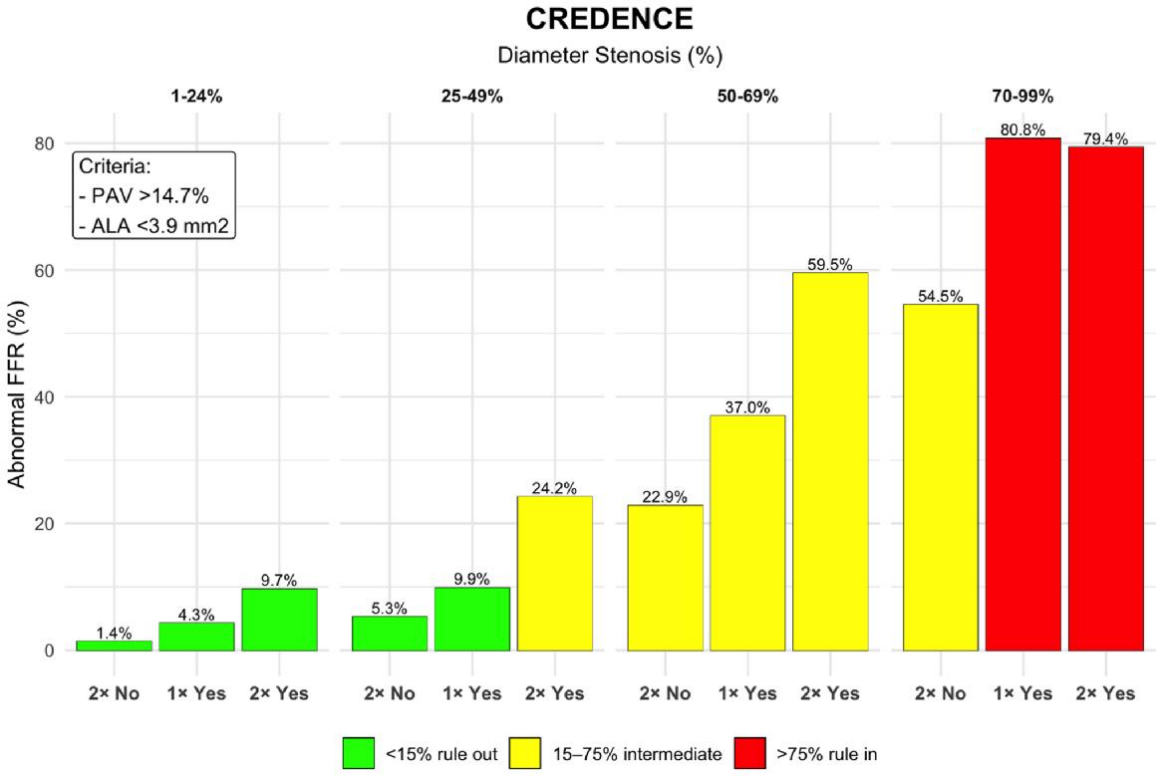
Diameter stenosis, PAV, and ALA for ruling-in or ruling-out abnormal fractional flow reserve (FFR) in CREDENCE study population. Vessels are grouped by diameter stenosis categories and further stratified by PAV and ALA cut-off values. ‘2× Yes’ denotes vessels with both PAV >14.7% and ALA <3.9 mm^2^, ‘1× Yes’ indicates vessels meeting one of these criteria, and ‘2× No’ indicates vessels with PAV <14.7% and ALA >3.9 mm^2^. Green bars represent vessels confidently ruled-out (<15% abnormal FFR), red bars represent vessels ruled-in (>75% abnormal FFR), and yellow bars denote intermediate-risk vessels (15–75% abnormal FFR).

**Figure 5:**
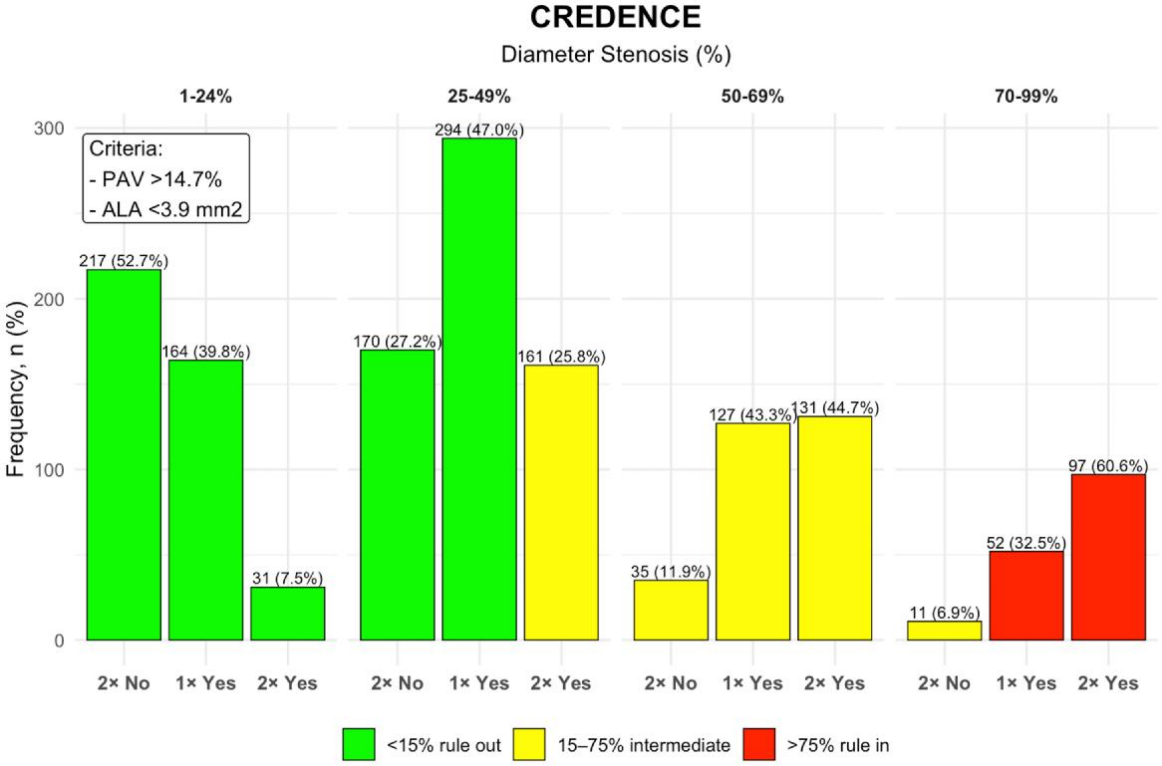
Frequency of vessels in each rule-in and rule-out category based on diameter stenosis, PAV, and ALA in the CREDENCE study population. This histogram shows the number of vessels stratified by diameter stenosis categories (1–24%, 25–49%, 50–69%, and 70–99%) and classified according to PAV and ALA criteria. ‘2× Yes’ indicates vessels meeting both criteria (PAV >14.7% and ALA <3.9 mm^2^), ‘1× Yes’ indicates vessels meeting one criterion, and ‘2× No’ indicates neither. Bar colours represent abnormal fractional flow reserve (FFR) risk categories: green (<15% rule-out), yellow (15–75% intermediate risk), and red (>75% rule-in).

Application of the three-metric framework, integrating diameter stenosis with PAV (>14.7%) and ALA (<3.9 mm^2^), enabled detailed risk stratification within each stenosis category. In CREDENCE, all vessels with 1–24% stenosis were successfully ruled-out for ischaemia, with an abnormal FFR prevalence <15% threshold irrespective of plaque features. For the 25–49% stenosis group, 74% of vessels were also ruled-out; however, the remaining 26% with high-risk features (large PAV and small ALA) were classified as intermediate risk with a 24% ischaemia prevalence. All vessels with 50–69% stenosis were intermediate risk, with ischaemia prevalence varied from 23% in vessels with small PAV and large ALA area to 60% in vessels with large PAV and small ALA. Finally, among severely stenotic vessels (70–99%), 93% were confidently ruled-in, with ischaemia prevalence exceeding 75%. The remaining 7%, which had small PAV and large ALA, had an ischaemia rate of 54.5% and were thus considered intermediate risk. *Figures 4* and *5* summarize the rule-in and rule-out categories.

**Figure 6:**
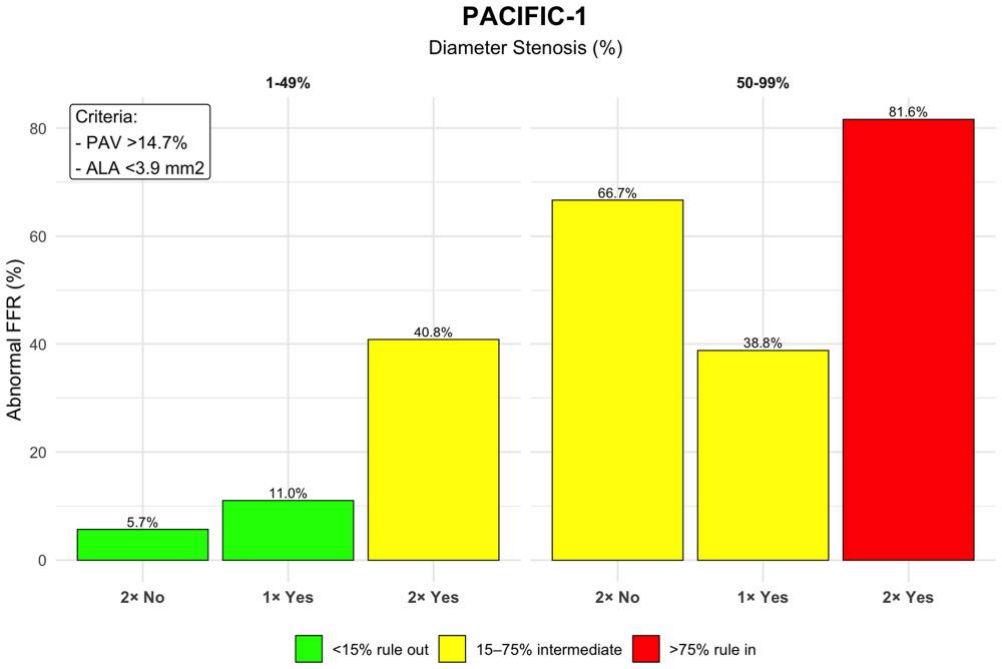
Diameter stenosis, PAV, and ALA for ruling-in or ruling-out abnormal fractional flow reserve (FFR) in PACIFIC-1 study population. Vessels are grouped by diameter stenosis categories and stratified using PAV and ALA cut-offs. ‘2× Yes’ denotes vessels meeting both criteria (PAV >14.7% and ALA <3.9 mm^2^), ‘1× Yes’ denotes vessels meeting one criterion, and ‘2× No’ denotes neither. Green bars indicate vessels confidently ruled-out (<15% abnormal FFR), red bars indicate vessels ruled-in (>75% abnormal FFR), and yellow bars represent intermediate-risk vessels (15–75% abnormal FFR).

**Figure 7:**
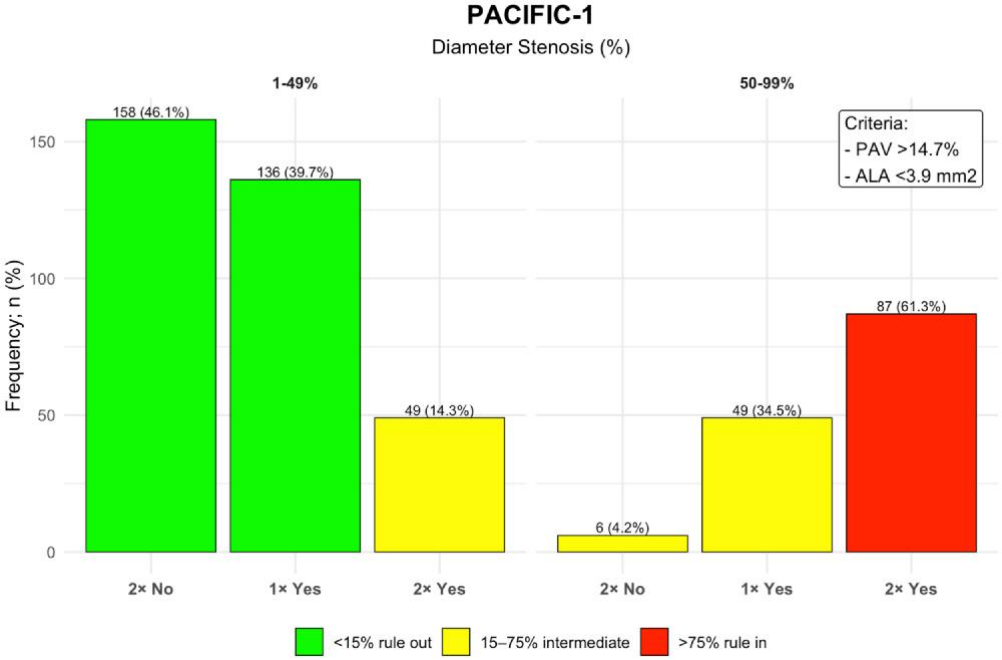
Frequency of vessels in each rule-in and rule-out category based on diameter stenosis PAV, and ALA in the PACIFIC-1 study population. This histogram shows the number of vessels stratified by diameter stenosis categories (1–49% and 50–99%), PAV and ALA cut-offs. ‘2× Yes’ indicates vessels meeting both criteria (PAV >14.7% and ALA <3.9 mm^2^), ‘1× Yes’ indicates vessels meeting one criterion, and ‘2× No’ indicates neither. Bar colours represent abnormal FFR risk categories: green (rule-out, <15% abnormal FFR), yellow (intermediate risk, 15–75%), and red (rule-in, >75%).

In the PACIFIC-1 cohort, 86% of vessels with non-obstructive disease (1–49% stenosis) were ruled-out. The remaining 14%, characterized by high-risk features (large PAV and small ALA), were classified as intermediate risk, with a 41% prevalence of ischaemia. Among vessels with obstructive disease (50–99% stenosis), the framework ruled-in 61% of vessels; this subgroup, defined by the same high-risk plaque features, had an ischaemia prevalence of 82%, while the remaining 39% were classified as intermediate risk (*Figures 6* and *7*). Exploratory vessel-level analyses applying the same stenosis categories as used in the CREDENCE cohort (1–24%, 25–49%, 50–69%, and 70–99%) were also performed and are presented in Supplementary data online, *Figure S1A* and *B*.

### Sensitivity analysis with PAV and ALA thresholds by Youden’s index

Thresholds were also derived using Youden’s index in the CREDENCE cohort (16.7% for PAV, 3.8 mm^2^ for ALA) and compared with median values (14.7% and 3.9 mm^2^, respectively). Diagnostic performance did not differ significantly between the median and Youden-derived thresholds in either cohort: CREDENCE (AUC 0.76 vs. 0.75; *P* = 0.116) and PACIFIC-1 (AUC 0.81 vs. 0.82; *P* = 0.882). The Youden-derived cut-offs yielded similar ischaemia risk categories to those obtained using median thresholds (see Supplementary data online, Figure 2*A*-*C*).

## Discussions

This study demonstrates that a simplified three-metric framework, integrating AI-guided quantitative CT parameters of diameter stenosis, PAV, and ALA, can effectively stratify ischaemia risk. By applying clinically relevant thresholds for PAV (14.7%) and ALA (3.9 mm^2^) alongside stenosis severity, the framework enables confident rule-out in most non-obstructive vessels and reliable rule-in in the majority of high-grade stenoses. Building on prior CREDENCE and PACIFIC-1 analyses^12^ which reported associations between quantitative plaque and lumen features and myocardial ischaemia, our study represents a novel advancement by defining clinically relevant thresholds for PAV and ALA and validating their application within a simplified, structured rule-in/rule-out framework to support clinical decision-making.

### Quantitative CT for ischaemia assessment

With rising healthcare costs and limited resources, optimizing the use of diagnostic tests is increasingly important. Although guidelines now recommend CCTA as the first-line test for ruling-out CAD,^1,22^ interpretation still relies heavily on stenosis severity. The finding of an obstructive lesion often triggers additional functional tests, an approach with limited effectiveness and increased healthcare costs.^14^ Our findings shows that adding quantitative CCTA parameters such as PAV and ALA to stenosis severity offers a more refined method for ischaemia risk assessment.

AI-QCT enables automated extraction of numerous quantitative parameters, including plaque composition, plaque burden and vessel characteristics. For this study, we focused on PAV and ALA due to their well-established, independent association with ischaemia and their suitability for clinical application.^13,24,25^ PAV reflects total plaque burden and correlates with ischaemia even in vessels with non-obstructive or intermediate stenosis,^10,26^ likely through effects on endothelial dysfunction, inflammation, and microvascular impairment.^27–29^ Similarly, ALA, as one of absolute quantitative lumen parameters, have been shown to predict functionally significant stenoses.^30^ Our findings confirm that combining these metrics with stenosis severity enhances diagnostic performance for ischaemia detection.

### Risk stratification in non-obstructive CAD

One key finding of this study is the framework’s ability to refine risk stratification in non-obstructive CAD. In the CREDENCE cohort, all vessels with 1–24% stenosis could be ruled-out for ischaemia given their consistent prevalence of abnormal FFR <10%. Further diagnostic testing in these patients is likely unnecessary, consistent with prior studies showing favourable prognosis in minimal stenosis and very low myocardial infarction rates.^1,23^

Within the 25–49% stenosis group, 74% of vessels in CREDENCE met rule-out criteria, which was confirmed in the PACIFIC-1 cohort, where 86% of vessels with 1–49% stenosis were also ruled-out. However, the model also identified a clinically relevant subset (26% in CREDENCE and 14% in PACIFIC-1) that, despite being non-obstructive, were at an intermediate risk for ischaemia due to adverse plaque and lumen characteristics (large PAV and small ALA). These observations highlight the added value of quantitative plaque analysis beyond anatomical assessment, enabling the detection of at-risk patients with non-obstructive disease who might otherwise be falsely reassured, and who may, depending on the clinical context, benefit from additional functional testing or more intensive preventive therapy.

The overall prevalence of abnormal FFR in vessels with 25–49% stenosis was 12% in CREDENCE and 12.8% in PACIFIC-1 (vessels 1–49% stenosis) consistent with prior studies. Park *et al*. reported that nearly 20% of lesions with <50% stenosis were ischaemic,^24^ while another study identified ischaemia in 7% of vessels without obstructive lesions.^31^ Collectively, these findings highlight that reliance on anatomical severity alone may underestimate ischaemia risk in a clinically relevant subset of patients.

### Intermediate and obstructive stenoses

For vessels with 50–69% stenosis, our framework did not yield definitive rule-in or rule-out classification and thus categorized them as intermediate risk. However, it still provided valuable stratification, with ischaemia prevalence ranging from 23% in the lowest-risk subgroup (small PAV, large ALA) to 60% in the highest-risk subgroup (large PAV, small ALA). These distinctions support a patient-tailored approach: for example, in a patient with atypical symptoms and a single lesion with low-risk quantitative features, additional functional testing may offer limited incremental value and conservative management could be appropriate, whereas those with high-risk features or multi-vessel involvement might warrant additional testing to guide revascularization. Although not directly evaluated in this study, this approach may be a practical strategy in clinical practice.

The framework was more definitive for lesions with 70–99% stenoses, where 93% of vessels were ruled-in for ischaemia. Even so, a small subset with low PAV and large ALA remained at intermediate risk, underscoring that not all severe stenoses are functionally equivalent. The current findings from PACIFIC-1 reinforced these results, with 61% of vessels with 50–99% stenosis meeting rule-in criteria, further supporting the framework’s utility in identifying functionally significant disease within anatomically obstructive lesions.

### Clinical integration and advantages

Many studies have shown that quantitative CCTA features predict ischaemia and adverse events.^11,32^ However, clinical adoption requires clearly defined thresholds for these metrics, where the post-test probability meaningfully alters patient management. Notably, the 14.7% PAV threshold identified in our study aligns closely with the 12.2% threshold reported by Kero *et al*.,^33^ in a similar large cohort, supporting its potential as a robust marker for ischaemia risk, although further validation is needed to harmonize thresholds across different software platforms.

Within current clinical CCTA reporting standards, including CAD-RADS 2.0,^21^ plaque assessment is primarily based on visual or semi-quantitative interpretation and may be subject to inter-observer variability.^34^ In this context, AI-guided quantitative CT analysis provide automated, objective, and reproducible quantitative plaque metrics, that may improve risk stratification. The present study was not designed to compare or replace CAD-RADS–based management pathways, but to evaluate whether such quantitative metrics may support ischaemia risk stratification. When used alongside existing CCTA reporting standards, this approach may support a standardized interpretation of ischaemia risk.

Finally, this simplified framework can be integrated into routine clinical workflow, with AI-QCT analysis performed directly after CCTA acquisition to generate standardized quantitative metrics, including diameter stenosis, plaque burden (PAV), and ALA. When interpreted alongside stenosis severity, these parameters provide clinicians with an additional layer of information to support ischaemia risk assessment, classifying vessels as rule-in, rule-out, or intermediate risk in a consistent and structured manner. In settings where more advanced machine learning models such as AI-QCT_ISCHAEMIA_ (which incorporates 37 AI-QCT parameters) are unavailable, this simplified framework may serve as a practical and accessible tool to enhance ischaemia risk stratification in patients with suspected CAD.^12^

### Limitations

This study represents a *post-hoc* analysis of two cohorts (CREDENCE and PACIFIC-1). Although findings were consistent across both datasets, prospective validation in larger and more diverse populations is needed to confirm generalizability. The reference standard for ischaemia in this study was invasive FFR, which primarily reflects epicardial vessel-level physiology and may not fully capture other contributors to ischaemia, such as microvascular dysfunction. Future studies incorporating complementary non-invasive functional imaging modalities as additional reference standards may provide further insight.

The quantitative parameters used in this framework were derived using a single AI-guided plaque quantification software, which may limit applicability to other platforms with different image processing algorithms. Thresholds for PAV and ALA should therefore be interpreted cautiously and may require calibration when applied to alternative software systems. Additionally, while this study focused on ischaemia risk stratification, it was not designed to evaluate long-term clinical outcomes or the impact of this framework on downstream patient management. These aspects should be addressed in future prospective studies.

## Conclusions

This study demonstrates that a simplified framework combining three quantitative CCTA metrics (diameter stenosis, PAV and ALA) can enhance myocardial ischaemia risk stratification. Applying clinically relevant thresholds for PAV (14.7%) and ALA (3.9 mm^2^), enables confident rule-out of ischaemia in most vessels with non-obstructive disease and reliable rule-in among those with high-grade stenosis. Importantly, it also identifies a subset of non-obstructive lesions with high-risk plaque and lumen features that may warrant further evaluation. These findings support the potential integration of quantitative plaque and lumen assessment into routine CCTA interpretation to improve functional risk assessment of CAD.

## Supplementary Material

jeag094_Supplementary_Data

## Data Availability

The data underlying this article will be shared on reasonable request from the corresponding author.

## Author information

**CREDENCE and PACIFIC-1 Investigators**: Ran Heo, MD, PhD (Division of Cardiology, Department of Internal Medicine, Hanyang University Medical Center, Seoul, Korea), Hyung-Bok Park, MD (Division of Cardiology, Department of Internal Medicine, International St. Mary's Hospital, Catholic Kwandong University College of Medicine, Incheon, South Korea), Hugo Marques, MD, PhD (Faculdade de Medicina da Universidade Católica Portuguesa, Lisboa, Portugal), Wijnand J. Stuijfzand, MD, PhD (Department of Cardiology, Amsterdam UMC, Vrije Universiteit Amsterdam, Amsterdam, The Netherlands), Jung Hyun Choi, MD, PhD (Department of Cardiology, Pusan National University Hospital, Busan, South Korea), Joon-Hyung Doh, MD (Division of Cardiology, Inje University Ilsan Paik Hospital, South Korea), Ae-Young Her, MD, PhD (Division of Cardiology, Department of Internal Medicine, Kangwon National University, College of Medicine, Kangwon National University School of Medicine, Chuncheon, South Korea), Bon-Kwon Koo, MD, PhD (Department of Internal Medicine, Seoul National University Hospital, Seoul, South Korea), Chang-Wook Nam, MD, PhD (Cardiovascular Center, Keimyung University Dongsan Hospital, Daegu, South Korea), Sang-Hoon Shin, MD (Division of Cardiology, Department of Internal Medicine, Ewha Women's University Seoul Hospital, Seoul, South Korea), Jason Cole, MD (Mobile Cardiology Associates, Mobile, Alabama, USA), Alessia Gimelli, MD (Department of Imaging, Fondazione Toscana Gabriele Monasterio, Pisa, Italy), Muhammad Akram Khan, MD (Cardiac Center of Texas, McKinney, Texas, USA), Bin Lu, MD (State Key Laboratory of Cardiovascular Disease, Fuwai Hospital, Beijing, China), Yang Gao, MD (State Key Laboratory of Cardiovascular Disease, Fuwai Hospital, Beijing, China), Faisal Nabi, MD (Houston Methodist Hospital, Houston, Texas, USA), Mouaz H. Al-Mallah, MD (Houston Methodist Hospital, Houston, Texas, USA), Ryo Nakazato, MD (Cardiovascular Center, St. Luke's International Hospital, Tokyo, Japan), Randall C. Thompson, MD (Saint Luke's Mid America Heart Institute, Kansas, Missouri, USA), James J. Jang, MD (San Jose Medical Center, Kaiser Permanente, San Jose, CA, USA), Michael Ridner, MD (University of Alabama-Birmingham, Birmingham, Alabama, USA), Chris Rowan, MD (University of Wisconsin-Madison, Wisconsin, USA), Erick Avelar, MD (Oconee Heart and Vascular Center, University of GA School of Medicine, Athens, GA, USA), Philippe Généreux, MD (Morristown Medical Center, New Jersey, USA), Guus A. de Waard, MD (Department of Cardiology, Amsterdam UMC, Vrije Universiteit Amsterdam, Amsterdam, The Netherlands)
