# Supplementary figures and images for: Artificial intelligence-guided quantitative coronary CT assessment to rule-in or rule-out myocardial ischaemia
Source: Eur Heart J Cardiovasc Imaging. 2026 Apr 13;27(6):1192–204. doi: 10.1093/ehjci/jeag094 (PMC13222718; doi:10.1093/ehjci/jeag094)

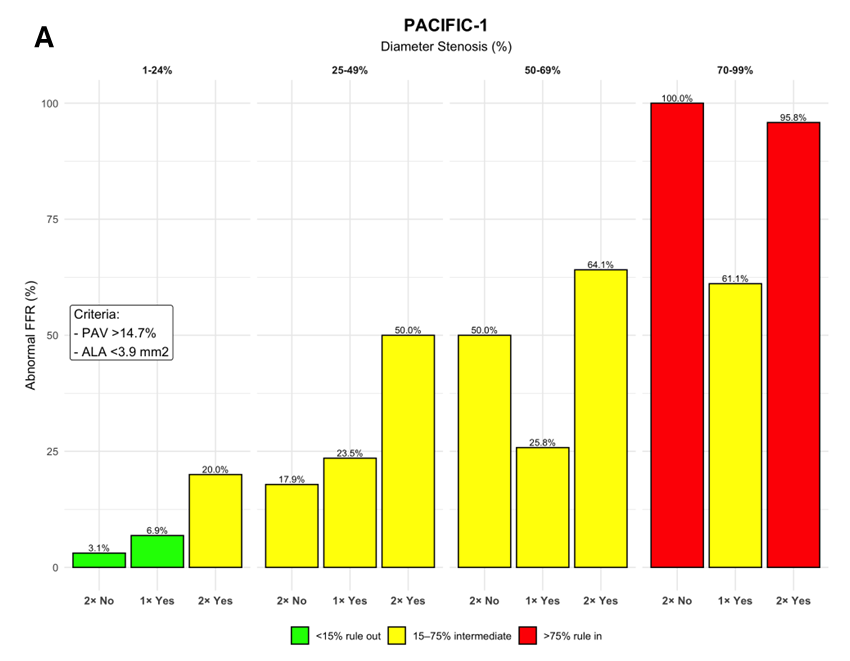

Supplement: jeag094_Supplementary_Data [file jeag094_supplementary_data.zip › Supplementary Figure 1A.PNG]

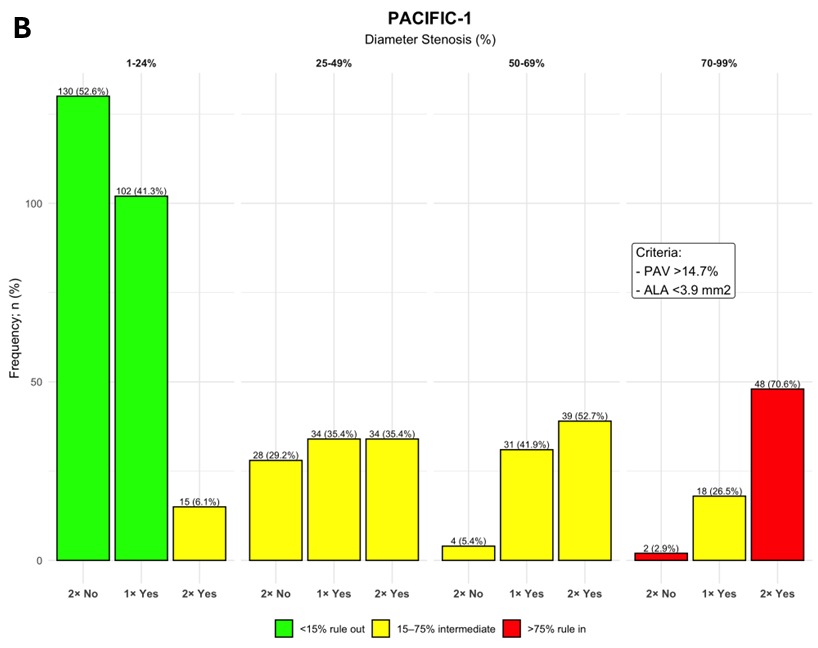

Supplement: jeag094_Supplementary_Data [file jeag094_supplementary_data.zip › Supplementary Figure 1B.PNG]

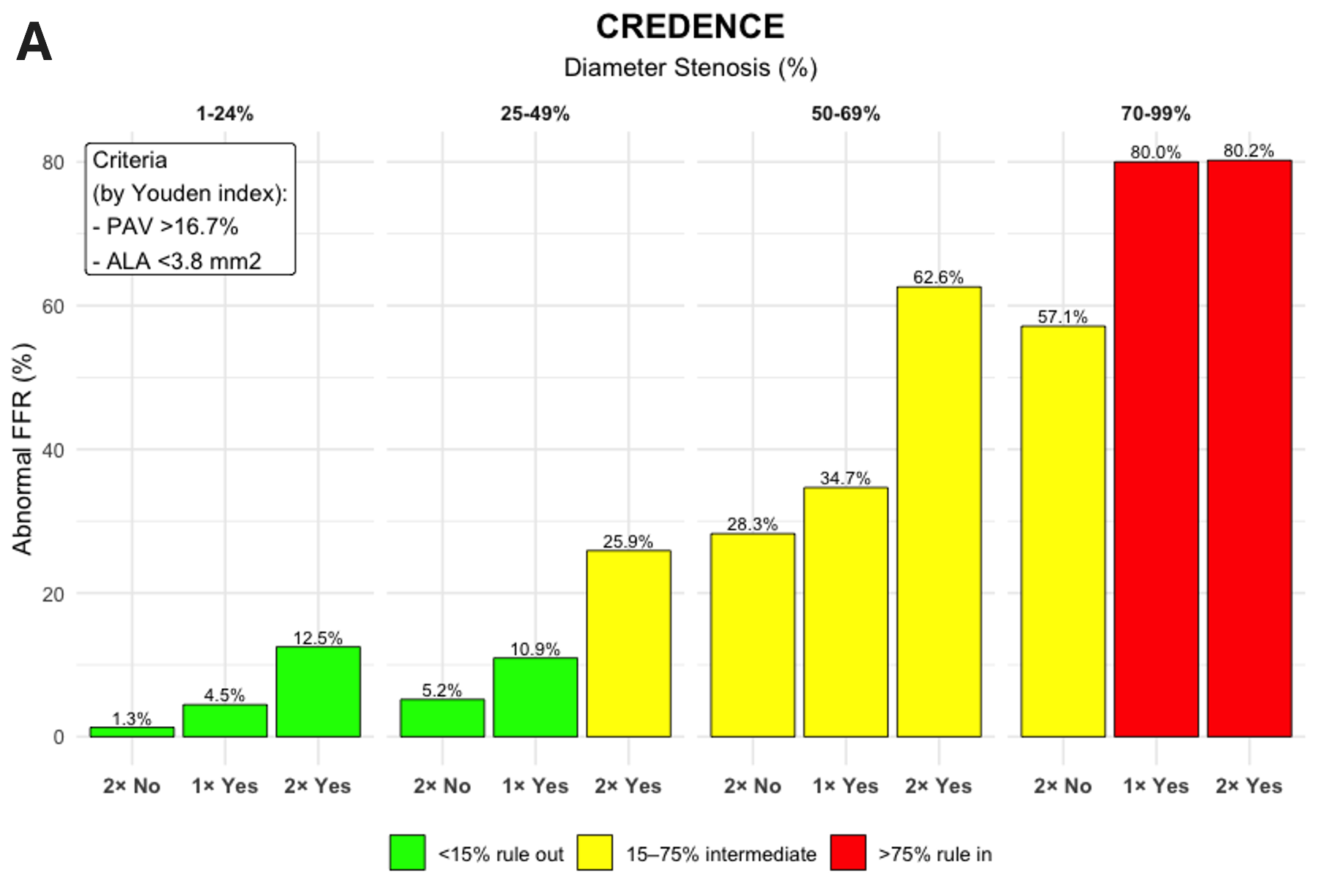

Supplement: jeag094_Supplementary_Data [file jeag094_supplementary_data.zip › Supplementary Figure 2A.png]

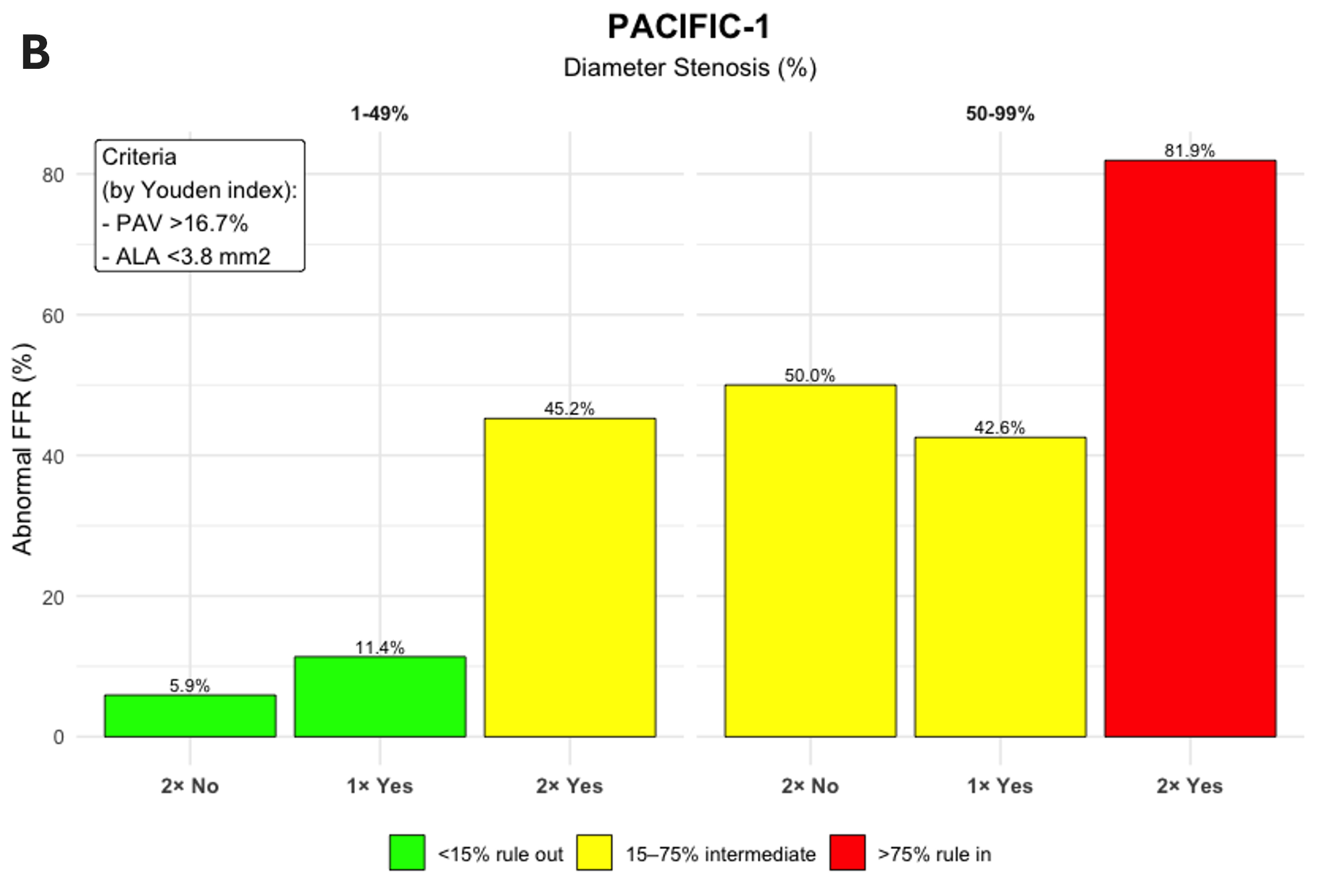

Supplement: jeag094_Supplementary_Data [file jeag094_supplementary_data.zip › Supplementary Figure 2B.png]

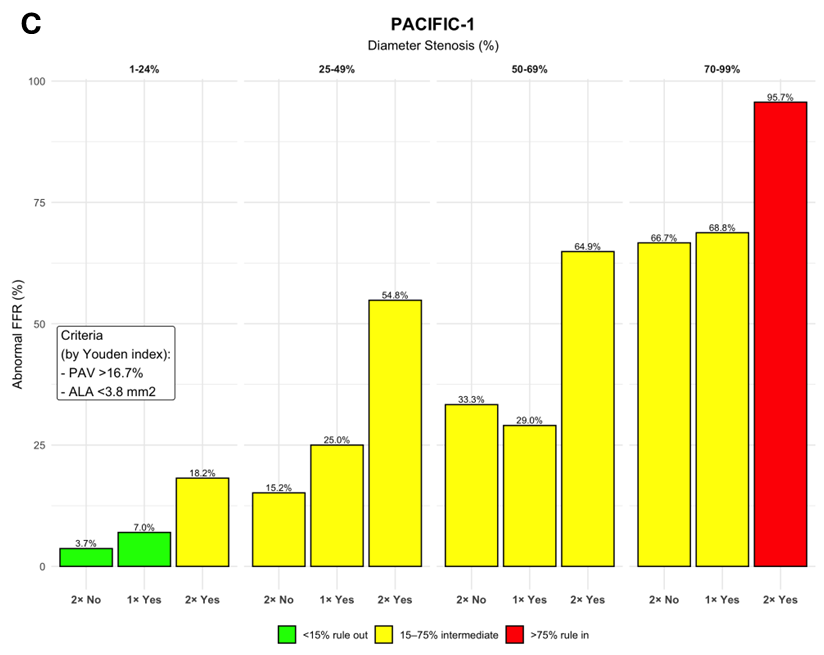

Supplement: jeag094_Supplementary_Data [file jeag094_supplementary_data.zip › Supplementary Figure 2C.PNG]
